# Bidirectional and reversible tuning of the interlayer spacing of two-dimensional materials

**DOI:** 10.1038/s41467-021-26139-5

**Published:** 2021-10-07

**Authors:** Yiran Ding, Mengqi Zeng, Qijing Zheng, Jiaqian Zhang, Ding Xu, Weiyin Chen, Chenyang Wang, Shulin Chen, Yingying Xie, Yu Ding, Shuting Zheng, Jin Zhao, Peng Gao, Lei Fu

**Affiliations:** 1 The Institute for Advanced Studies, Wuhan University, Wuhan, 430072 China; 2 College of Chemistry and Molecular Sciences, Wuhan University, Wuhan, 430072 China; 3 Department of Physics, University of Science & Technology of China, Hefei, 230026 China; 4 Electron Microscopy Laboratory, School of Physics, Peking University, Beijing, 100871 China

**Keywords:** Nanoscale materials, Two-dimensional materials

## Abstract

Interlayer spacing is expected to influence the properties of multilayer two-dimensional (2D) materials. However, the ability to non-destructively regulate the interlayer spacing bidirectionally and reversibly is challenging. Here we report the preparation of 2D materials with tunable interlayer spacing by introducing active sites (Ce ions) in 2D materials to capture and immobilize Pt single atoms. The strong chemical interaction between active sites and Pt atoms contributes to the intercalation behavior of Pt atoms in the interlayer of 2D materials and further promotes the formation of chemical bonding between Pt atom and host materials. Taking cerium-embedded molybdenum disulfide (MoS_2_) as an example, intercalation of Pt atoms enables interlayer distance tuning via an electrochemical protocol, leading to interlayer spacing reversible and linear compression and expansion from 6.546 ± 0.039 Å to 5.792 ± 0.038 Å (~11 %). The electronic property evolution with the interlayer spacing variation is demonstrated by the photoluminescence (PL) spectra, delivering that the well-defined barrier between the multilayer and monolayer layered materials can be artificially designed.

## Abstract (web summary)

Modifying the interlayer spacing in two-dimensional materials is an effective approach to tune their chemical and physical properties. Here the authors report that intercalation of Pt atoms into cerium-doped MoS_2_ enables adjusting the interlayer spacing bidirectionally and reversibly.

## Introduction

Recent studies on two-dimensional (2D) materials have unveiled various fascinating chemical and physical properties that are dramatically different from their bulk counterpart^1–5^. These unique properties endow 2D materials with a wide range of applications in electronic, optical, thermal, and energy conversion devices^6–8^. 2D materials exhibit remarkable interlayer coupling across the layers in nanoscale^9,10^, which is mainly manifested on the layer-dependent bandgap^11,12^, intralayer electronic, and orbital structures^13,14^. Therefore, manipulating the interlayer interaction of 2D materials opens a new field of material property engineering. The general approaches include modulating layer number^15^, changing the interlayer stacking configuration^16,17^, adjusting the concentration of electron and hole^18^ and inducing the structure lattice distortion^19,20^. However, current strategies for controlling the interlayer interaction are typically flawed in unavoidable structural transformations of host materials or are incapable to maintain the stability of interlayer spacing without the constant external stimulation in physical strategy. Recently, interlayer spacing regulation methods were increasingly studied as effective approaches to tune the chemical and physical properties of 2D materials without destroying the intrinsic in-plane structure of 2D materials^21–23^, such as chemical intercalation to widen the interlayer spacing^24–26^ or physical compression to realize the shortening^20,27^. However, all those works only can achieve the monotonous unidirectional adjustment of the interlayer spacing of 2D materials. To the best of our knowledge, the ability to non-destructively regulate the interlayer spacing of 2D materials in a freewheeling way, i.e., bidirectionally and reversibly, has never been achieved.

In this work, we report a 2D material with highly tunable interlayer spacing by the elaborate presetting of active diatomic (A‒B) pairs for establishing the reversible chemical bonding in the interlayer of material. Such a flexible layered structure is demonstrated by Pt, Ce**‒**MoS_2_ as an example, in which single-atom Pt is effectively stabilized near the Ce sites pre-embedded in MoS_2_. By intercalating and eluting the monodispersed Pt atoms through an electrochemical method, the interlayer spacing of Ce−MoS_2_ can be reversibly compressed and expanded from 6.546 ± 0.039 Å to 5.792 ± 0.034 Å (~11.52%). The successfully bidirectional manipulation of the interlayer spacing of MoS_2_ facilitates the effective tuning of the interlayer coupling, leading to the controlling of their optoelectronic property in a dynamical and reversible way. The interlayer spacing regulation strategy will significantly weaken the dependence of material properties on the number of layers, leading to the property crossover between multilayer and few-layer MoS_2_.

## Results and discussion

### Interlayer spacing regulation of 2D MoS_2_

**Fig. 1:**
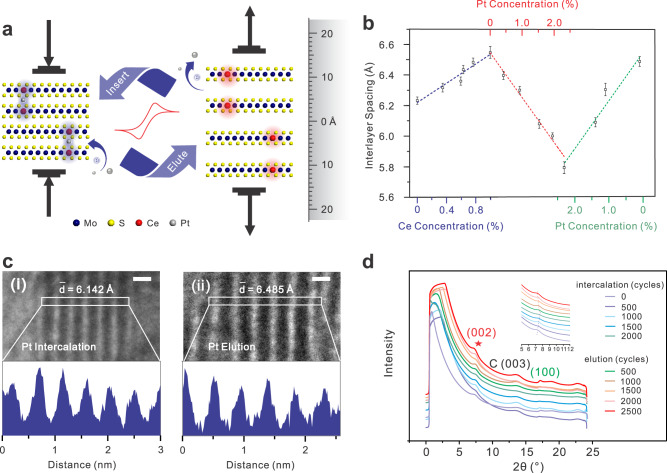
Bidirectional continuous interlayer spacing regulation of Pt, Ce–MoS_2_. **a** Schematic diagram of 2D Pt, Ce−MoS_2_ interlayer spacing regulation. With Pt inserting and eluting, the chemical bonds are formed reversibly (indicated by the blue or red shadow areas), resulting in the compression and expansion of the interlayer spacing (indicated by the arrow direction). **b** Interlayer spacing regulation of MoS_2_ via Ce doping (blue dashed line), Pt intercalating (red dashed line), and Pt eluting (green dashed line). Error bars represent standard deviation over 26 independent replicates at least. **c** TEM images of the layered edges of Pt-intercalated and Pt-eluted MoS_2_ and the intensity profiles in the bottom panels corresponding to the areas marked by white rectangles in the upper panels. **d** means the average interlayer spacing, which is obtained from the average distance between two neighboring peaks in the intensity profiles. Scale bar, 5 Å. **d** In situ SRXRD spectra of MoS_2_ with Pt intercalating and Pt eluting.

We first doped Ce with different concentrations into parallel MoS_2_ samples to enlarge the interlayer spacing of MoS_2_ to varying degrees, which offers active sites to anchor and stabilize the monodispersed Pt atoms (Seen in Method). The interlayer spacing of MoS_2_ exhibits a linear increase with the Ce concentration owing to the gradual weakening of interlayer coupling caused by the electron that transferred to the S–Mo anti-bonding orbitals^28^, as shown in Fig. 1a. Each of these interlayer spacing values is derived from the average value of data collected from 30 different locations within a sample (Supplementary Fig. 1). When the concentration of Ce increases from 0% to 0.995% (controlled by the concentration of the precursor), the interlayer spacing of MoS_2_ changes from 6.232 ± 0.024 Å to 6.546 ± 0.039 Å. The monodispersed Pt atoms were intercalated into the interlayer of Ce-activated MoS_2_ via an electrochemical process to reduce the interlayer spacing (Seen in Method). It is contrary to the situation in the reported cases, in which the chemical intercalation of metal atoms usually leads to the opening of the interlayer spacing^25,29–31^. When the concentration of Pt increases from 0% to 2.31% (controlled by the cycle number during the cyclic voltammetry (CV) process), the interlayer spacing of MoS_2_ changes from 6.546 ± 0.039 Å to 5.792 ± 0.038 Å (Fig. 1a and Supplementary Fig. 2). Relative to the intrinsic value (6.232 ± 0.024 Å), the variation range of the interlayer spacing can be from 5.04% to −7.06%. It is worth mentioning that the interlayer spacing can be compressed below the value of intrinsic MoS_2_. To the best of our knowledge, it is realized by the intercalation for the first time. The single Pt atoms that anchor on Ce‒MoS_2_ can be electrochemically eluted (Seen in Method) and the interlayer spacing will get expanded. When the concentration of Pt decreases from 2.31% to 0.14% (controlled by the cycle number during the CV process), the interlayer spacing gets recovered to 6.494 ± 0.032 Å (Fig. 1a and Supplementary Fig. 3). The good linear reversible relationship presents that the 2D MoS_2_ can stretch out and drawback along an axis perpendicular to the planes with the migration and immigration of the monodispersed Pt atoms. Figure 1c exhibits the typical edge structure of Pt-intercalated and Pt-eluted MoS_2_ by transmission electron microscopy (TEM), from which we can get the interlayer spacing information. In Fig. 1d, in situ synchrotron radiation X-ray diffraction (SRXRD) further demonstrated the evolution of the interlayer spacing with the variation of Pt concentration during the electrochemical intercalation and elution process. The (002) peak of MoS_2_ (characteristic peak of interlayer spacing) continuously deviates to a high angle during the intercalation process, indicating the interlayer spacing is shrinking. Contrarily, the (002) peak moves to a lower angle with the increase of the cycle number in the elution process, which is proof that Pt elution results in the interlayer spacing extending. The corresponding CV curves are shown in Supplementary Fig. 4. Therefore, our diatomic pair strategy regulates the interlayer spacing of 2D material bidirectionally and reversibly.

### Structure characterization of 2D Pt, Ce–MoS_2_

**Fig. 2:**
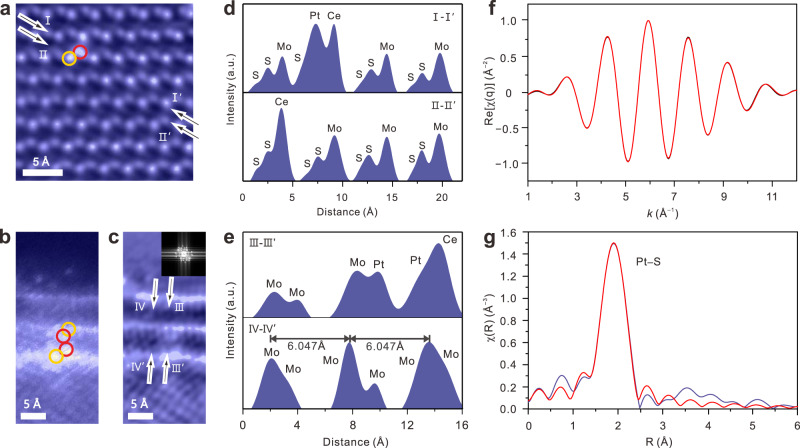
Structure characterizations of Pt, Ce–MoS_2_. **a** Corrected ADF–STEM image of Pt, Ce–MoS_2_. **b** As recorded and **c** corrected cross-sectional ADF–STEM images of Pt, Ce–MoS_2_ at the edge site. Yellow circle: Ce atoms and Red circle: Pt atoms. Arrow: selected area corresponding to the line intensity profile shown in **e**. **d**, **e** Line intensity profiles in the selected regions in image **a** and **c** along I‒Iʼ, II‒IIʼ, III‒IIIʼ, IV‒IVʼ. **f** Pt *L*_3_-edge EXAFS fitting results of Pt, Ce–MoS_2_ in q space. **g** *k*^2^-weight Fourier transformation of Pt *L*_3_-edge EXAFS. The EXAFS oscillations were fitted according to single-atom scattering equation, using the FEFF models.

For illuminating the role of Ce and Pt played in the interlayer spacing regulation, high-angle annular dark-field scanning TEM (HAADF–STEM) was employed to clarify the 2D Pt, Ce–MoS_2_ structure. Except for the hexagonal rings of the MoS_2_ structure, there are several monodispersed atoms with their intensity stronger than that of Mo atoms (Fig. 2a, b and Supplementary Fig. 5, 6). Those brighter atoms can be divided into two types: the atoms that occupy Mo site marked with yellow circles and the other atoms that lie near S site marked with red circles. The statistical distributions of the ADF peak intensity corresponding to the brighter atoms marked with yellow circles and the Mo atoms are shown in Supplementary Fig. 7. Their intensity ratio is consistent with the value of Z_Ce_^1.7^/Z_Mo_^1.7^ (1.73). Therefore, those brighter heteroatoms on Mo sites are thought to be Ce^32^. Considering that the intercalated Pt atoms can probably be both above or below the MoS_2_ plane, the atoms in red circles with randomly distributed intensity could probably be Pt. Notably, it is found that Pt atoms usually existed in accompany with Ce atoms, implying their diatomic pair relationship. Furthermore, there are no Pt atoms near the Ce atoms after the elution process through the electrochemical method, indicating that the diatomic pair relationship can be built reversibly (Supplementary Fig. 8). And the structure of Ce–MoS_2_ can well remain when the Pt atoms were eluted from the interlayer of Ce–MoS_2_, which lays the foundation of reversible regulation of the 2D material interlayer spacing. The Ce–MoS_2_ endured five cycles of the repeated intercalation and elution process and remained its complete structure (Supplementary Fig. 9–11).

In order to further investigate the structural information of the 2D Pt, Ce−MoS_2_, extended X-ray absorption fine structure (EXAFS) measurements at Pt *L*_3_-edge were carried out (Fig. 2f, g) and the fitting results of the EXAFS spectra are displayed in Supplementary Table 1. There exists no Pt–Pt coordination bond, but only Pt–S coordination structure in the first coordination shell, which clearly demonstrates that Pt is intercalated in the form of a single atom into the MoS_2_ interlayer. X-ray photoelectron spectroscopy (XPS) spectra also confirm the existence of Pt–S but no Pt−Pt bond (Supplementary Fig. 12)^33^. In addition, TEM image and the related energy dispersive X-ray (EDX) spectroscopy mapping analysis also show that no aggregated Pt clusters form on MoS_2_ (Supplementary Fig. 13).

### Chemical environment variation in the interlayer spacing regulation of 2D MoS_2_

The variation of the chemical environment of MoS_2_ was studied for understanding the interlayer spacing regulation induced by the introduction of Ce and Pt atoms. The XPS of Ce–MoS_2_ suggests the electron transfer from Ce ions to MoS_2_ and the in-plane doping nature of Ce ions in MoS_2 _(Supplementary Fig. 14). The embedded Ce ions could weaken the interlayer coupling of adjacent MoS_2_ layers by injecting negative charges to the S–Mo anti-bonding and then change the stacking structure to enlarge the interlayer spacing of MoS_2_ planes (Supplementary Fig. 15, 16).

**Fig. 3:**
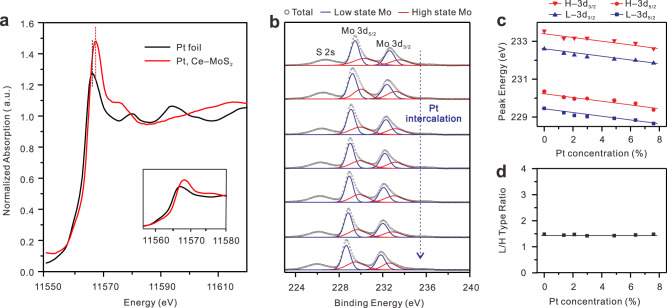
The variation of the chemical environment of MoS_2_ with the interlayer spacing variation. **a** The normalized XANES spectra at Pt *L*_3_ edge of the Pt, Ce–MoS_2_, and reference Pt foil. **b** XPS spectra of Mo 3d core level derived from the Pt, Ce–MoS_2_ samples with the increase of Pt concentration. **c** The peak position variation of Mo 3d with the increase of Pt concentration. H−3d_3/2_: high-state Mo 3d_3/2_; H−3d_5/2_: high-state Mo 3d_5/2_; L−3d_3/2_: low-state Mo 3d_3/2_; L−3d_5/2_: low-state Mo 3d_5/2_. **d** The ratio evolution of high-state Mo to low-state Mo with the increase of Pt concentration.

As shown in the X-ray absorption near edge structure (XANES) spectra (Fig. 3a), the absorption edge of Pt moves toward high energy (~1 eV), indicating that electrons are transferred from Pt to MoS_2_. The XANES result was consistent with the XPS analysis. As for the intercalation of Pt (Fig. 3b), owing to the lower electronegativity of Pt atoms, electrons can be transferred from Pt to the host MoS_2_, resulting in the downshift of Mo 3d-binding energy. XPS spectrum of core Mo 3d exhibits two different valence states owing to the intrinsic defects and impurities, which are high-state Mo and low-state Mo (Fig. 3b and Supplementary Fig. 14). Notably, the intercalated Pt atoms exert a negligible effect on the valence state components of Mo, further conforming to the intercalation nature of Pt atoms instead of doping (Fig. 3c, d). Whereas, intercalating Pt atoms into the Ce–MoS_2_ could enhance the interlayer interaction by forming chemical bonding of S–Pt in the interlayer gaps, even if Pt serves as an electron donor. All the interlayer spacing values are calculated by density functional theory (DFT) to verify the reliability of experimental data, which are listed in Supplementary Table 2.

### Mechanism of the interlayer spacing regulation of 2D MoS_2_

In our strategy, an atomically dispersed synergy couple Ce–Pt is the key, in which monodispersed Ce ions can not only serve as a lifting jack to open up the interlayer spacing but also a good anchor for stabilizing Pt single atoms. It has been demonstrated that Pt atoms were dissolved from the Pt foil counter electrode into the H_2_SO_4_ electrolyte during the electrochemical CV process. The dissolved Pt atoms are then redeposited back on the cathode^34,35^. The strong chemical interaction between Ce ions and Pt atoms provides us an accessible approach to capture and stabilize the dissolved Pt single atoms, which is demonstrated by the DFT calculations (Supplementary Table 3). Besides, the intercalation energy barrier is much smaller than that of the surface absorption, indicating that the redeposited Pt atoms are inclined to intercalate in the interlayer of MoS_2_. Supplementary Fig. 4 shows the polarization curves of Ce–MoS_2_ during the Pt intercalation process. The gradually increasing current within the voltage range of −0.4~0 V means that the hydrogen evolution reaction (HER) catalytic performance is increasing, indicating that Pt atoms successfully intercalate in MoS_2_, considering that Pt can serve as the active site of HER reactivity^36^. For highlighting the role of Ce, the direct intercalation of Pt into pure MoS_2_ was conducted. It can be found that the interlayer spacing of MoS_2_ gets expanded (Supplementary Fig. 17), consisting with the reported work^30^. On one hand, Pt atoms tend to aggregate into nanoparticles without the stabilization of Ce ions, in which the steric effect will result in the interlayer spacing expansion. On the other hand, metal atom intercalation will also lead to the expansion of the interlayer spacing inevitably owing to the introduction of free electrons that will increase Fermi energy levels and expand the band gaps^25^. For the elution process, we displaced the Pt foil by graphite rod electrode as the counter electrode. According to previous reports, the combination of Pt and host material will be weakened when Pt catalyst is used for HER reaction^37^. Furthermore, a new peak corresponding to the Pt−O coordination will appear (confirmed by XPS in Supplementary Fig. 18)^38^, which may be caused by the adsorption of oxygen-related groups in H_2_O or OH^−^^37^. Considering that the catalyst is working in an acid solution, the oxygen-related groups should be contributed by H_2_O. The existence of such an intermediate (Pt−OH_2_) may result in the formation of either H_2_ (Tafel process) or vacancies. The stronger Pt−O bonds (bond dissociation energies, 4.3 eV) in Pt-OH_2_ force the breaking of Pt−S bonds (bond dissociation energies, 4.14 eV), resulting in the formation of a Pt−OH_2_ leaving group, followed by its dissolution and the elution of Pt^39,40^. Furthermore, this Ce−Pt diatomic pair strategy can be used in other 2D materials, for example, WS_2_ (Supplementary Fig. 19–21). Through understanding the role of diatomic pairs, it is enlightened that the interlayer spacing of 2D materials can be reversibly regulated as long as suitable atomic pairs are constructed. For example, we used the Pd instead of Ce as the A-type atom. Similar to Ce, Pd can anchor and stabilize monodispersed Pt atoms (Supplementary Fig. 22–24)^41^. As expected, the interlayer spacing of MoS_2_ can also be reversibly tuned by Pd−Pt atomic pair.

### Evolution of the electronic structure of 2D MoS_2_ with a different interlayer spacing

**Fig. 4:**
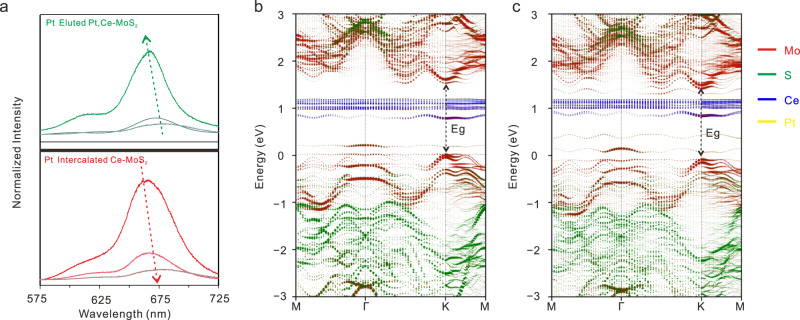
Electronic property evolution of 2D MoS_2_. **a** PL spectra of Ce–MoS_2_ with different interlayer spacing induced by the intercalating and eluting of Pt single atoms. Bottom, the PL spectrum evolution of Pt-intercalated Ce–MoS_2_ with Pt concentration increase. Top, the PL spectra evolution of Pt-eluted Pt, Ce–MoS_2_ with Pt concentration decrease. **b**, **c** calculated unfolded band structures of Ce−MoS_2_ **b** and Pt, Ce−MoS_2_ **c**. Eg, bandgap.

The evolution of the electronic structure of 2D MoS_2_ undergoing the interlayer spacing variation was explored. An interlayer-spacing dependent bandgap evolution derived from the PL spectra was observed, proving that the interlayer spacing can have a direct influence on the strength of interlayer interaction (Fig. 4a and Supplementary Fig. 25). The interlayer spacing of MoS_2_ increases with the Ce concentration, leading to the result that the PL of few-layer MoS_2_ evolves into monolayer-like MoS_2_^42^ (Supplementary Fig. 25). On the other hand, the interlayer coupling is enhanced when the interlayer spacing is reduced with the intercalating of Pt atoms. The main PL A peak position shifts from 663 nm to 681 nm with the significant attenuation of PL A peak intensity (Fig. 4a). Theoretical analysis has predicted the enhancement of the interlayer interaction with the approaching of the conduction and valence bands at the Fermi level and the aggrandizement of interband electronic relaxation weakens the PL intensity. Inversely, with the decreasing of Pt concentration in the elution process, the main PL A peak position shifts from 681 nm back to 667.5 nm and the PL A peak intensity becomes stronger, which indicates that the as-compressed MoS_2_ gets pulled. The calculated band structure further substantiated this theory, in which the bandgap of Ce–MoS_2_ is 1.648 eV whereas that value of Pt, Ce–MoS_2_ is 1.577 eV (Fig. 4b, c).

Considering that a certain amount of Ce and Pt atoms has been added to the systems and they can also affect the electronic structures. We calculated the bandgap (*E*_CBM_−*E*_VBM_) at the K point of intrinsic AA stacking MoS_2_ with the interlayer spacing variation (Supplementary Fig. 27). Compared with the AA stacking Ce–MoS_2_ with the same interlayer spacing, it can be deduced that both the Ce doping and interlayer spacing enlargement will lead to the increasing of the bandgap. Besides, we fixed the interlayer spacing of AA stacking Ce–MoS_2_ and AA stacking Pt, Ce–MoS_2_ and found that the Pt insertion would result in the decreasing of the bandgap (Supplementary Fig. 27). The calculation results further confirm the influence of interlayer spacing on the band structure of 2D materials^43^. The electronic property evolution of the 2D MoS_2_ with the interlayer spacing variation demonstrated by the PL spectra reveals that the dependency degree of the property of MoS_2_ on the layer number can be artificially designed, which brings down the barriers between single-layered and multilayered materials.

In conclusion, we achieved the bidirectional and reversible interlayer spacing tuning of a MoS_2_-based 2D material, realizing the regulation of materials fine structure and the property crossover between multilayer and few-layer MoS_2_. It can be expected that the flexible interlayer spacing tuning of 2D materials can inject new vitality to their property exploration and fundamental applications with precisely designed interlayer spacing.

## Methods

### Preparation of MoS_2_ nanosheets

MoS_2_ nanosheets were obtained by a hydrothermal reaction method. In all, 5 mmol L^−1^ (NH_4_)_6_Mo_7_O_24_·4H_2_O, 50 mmol L^−1^ CH_4_N_2_S and a certain amount of CeCl_3_ (dependent on the expected Ce-doping proportion, 0~10%) was dissolved in ultrapure water separately, followed by being mixed and stirred into a uniform solution. Then the mixed solution was then transferred into a high-pressure autoclave and was heated at 205 °C for 24 h. Ti foils, pretreated with nitric acid (HNO_3_) for 30 min, were set in the autoclave to serve as the growth substrates. The Ti foils covered by MoS_2_ nanosheets were collected and washed with deionized water several times. Intrinsic MoS_2_ nanosheets were synthesized without CeCl_3_ in the same method. For Pd-doping MoS_2_ nanosheets preparation, the Na_2_PdCl_4_ was employed as the dopant instead of CeCl_3_ and the other experimental parameters remained unchanged.

### Preparation of WS_2_ nanosheets

WS_2_ nanosheets were obtained by a hydrothermal reaction method. In all, 3 mmol L^−1^ (NH_4_)_6_H_2_W_12_O_40_·xH_2_O, 0.9 mol L^−1^ CH_3_CSNH_2_, 0.4 mol L^−1^ C_2_H_2_O_4_·2H_2_O, and a certain amount of CeCl_3_ (dependent on the expected Ce-doping proportion) were dissolved in ultrapure water separately, followed by being mixed and stirred into a uniform solution. Then the mixed solution was then transferred into a high-pressure autoclave and was heated at 200 °C for 24 h. Ti foils, pretreated with HNO_3_ for 30 min, were set in the autoclave to serve as the growth substrates. The Ti foils covered by WS_2_ nanosheets were collected and washed with deionized water several times.

### Compressing of the interlayer spacing of Ce–MoS_2_ via electrochemical intercalation of Pt

The electrochemical intercalation of Pt was performed based on the CV process in 1.0 M H_2_SO_4_ solution. A three-electrode system was installed using MoS_2_ nanosheets@Ti foil as the working electrode with a Pt foil as the counter electrode and a reversible hydrogen electrode (RHE) as the reference electrode. The RHE and Pt electrodes are bought from Gaoss Union Co., Ltd. (Wuhan, China). All the electrochemical processes were conducted based on a CHI610E electrochemical workstation (Chenhua, Shanghai, China). The system was continuously fed with 5 sccm hydrogen (H_2_) to exclude the oxygen (O_2_) produced by H_2_O oxidation in the platinum electrode. The electrochemical intercalation of Pt was performed by a CV process with the voltage from −0.4 V to 0 V vs. RHE at a scan rate of 100 mV s^−1^. The CV curves are shown in Supplementary Fig. 4. In such a CV process, the Pt foil at the anode will be slightly dissolved and redeposited onto the MoS_2_ nanosheets@Ti at the cathode, leading to the intercalation of the monodispersed Pt into the Ce‒MoS_2_ interlayer. The concentration of the monodispersed Pt intercalated into MoS_2_ interlayer was controlled by the cycle number of CV running segments.

### Extending of the interlayer spacing of Pt, Ce–MoS_2_ via electrochemical elution of Pt

In the process of electrochemical elution of Pt, the graphite rod electrode was employed as the counter electrode instead of Pt foil and the other experimental parameters remained unchanged. The CV curves are shown in Supplementary Fig. 4. The degree of the Pt eluting from MoS_2_ interlayer was controlled by the cycle number of CV running segments.

### Characterization

TEM characterizations were performed on high-resolution TEM (JEM–2100) operated at 200 kV. The TEM systems are coupled with an EDX system. HAADF–STEM images were obtained by a Nion U–HERMES200 operated at 60 kV. In situ SRXRD was performed at the BL14B1 beamline of Shanghai Synchrotron Radiation Facility (SSRF) with a wavelength of 0.8217 Å. The beam size at the sample was ~0.3 × 0.3 mm^2^ and was confined by vertical and horizontal slits. A MarCCD detector was used to acquire 2D X-ray diffraction signals with dwell time of 120 s. The signal is collected every 500 cycles during the CV process of intercalation and elution. PL spectroscopy was performed in a laser micro–Raman spectrometer (Renishaw in Via) with a laser excitation wavelength of 532 nm and a power of 1 mW. XPS measurements were conducted on a Thermo Scientific, ESCALAB 250Xi system. The C 1 s peak (284.8 eV) was used as the reference to calibrate the binding energies. Pt *L*_3_ edge (11564 eV) XAFS spectra were measured using the BL14W1 beamline at SSRF. The beamline was operated with a Si (111) double crystal monochromator and an uncoated glass mirror to reduce the higher harmonics component of the monochromatic beam. During the measurement, the synchrotron radiation ring was operated at 3.5 GeV and the current was maintained at 260 mA with a top-up mode. The samples were sealed in Kapton film with 10 mg loading. A Pt foil was used as a reference sample and measured in the transmission mode, whereas samples were measured in fluorescence mode, using the Lytle detector to collect the data. All XAFS spectra were processed using the IFEFFIT package.

### Calculations

Our DFT calculations employed the projector-augmented wave method^44^ as implemented in the Vienna ab initio simulation package^45,46^. The Perdew-Burke-Ernzerhol parametrization of the generalized gradient approximation was used for the exchange-correlation potential^47^. The plane-wave cutoff energy of 400 eV was used in all the calculations. To simulate the 1% Ce-doping, a 4 × 4 × 1 bilayer-MoS_2_ supercell was used, where a lattice constant of 3.190 Å was used for MoS_2_. In addition, a vacuum spacing of ~20 Å was used to prevent spurious interaction between periodic images. D2 method of Grimme was applied to take van der Waals interaction into account^48^. The Brillouin zone was sampled at G-point only during the geometry optimization. The energy and force are converged with the threshold of 10^−8^ eV and 2 meV/A, respectively. The unfolded band structures were calculated using the VaspBandUnfolding package.

## Supplementary information

Supplementary Information

## Data Availability

The data that support the findings of this study are available from the corresponding author upon request.
