# Supplementary material for: Bidirectional and reversible tuning of the interlayer spacing of two-dimensional materials
Source: Nat Commun. 2021 Oct 7;12:5886. doi: 10.1038/s41467-021-26139-5 (PMC8497624; doi:10.1038/s41467-021-26139-5)
Supplement: Supplementary file 1 — Supplementary Information [file 41467_2021_26139_MOESM1_ESM.pdf]

Supplementary Information

Bidirectional and reversible tuning of the interlayer spacing of two-dimensional materials

Yiran Ding[1†], Mengqi Zeng,[2†] Qijing Zheng[3], Jiaqian Zhang[2], Ding Xu[2], Weiyin Chen[2], Chenyang Wang[2], Shulin Chen[4], Yingying Xie[2], Yu Ding[2], Shuting Zheng[2], Jin Zhao[3], Peng Gao[4], and Lei Fu[1,2]*

[1]*The Institute for Advanced Studies, Wuhan University, Wuhan 430072, China*

[2]*College of Chemistry and Molecular Sciences, Wuhan University, Wuhan 430072, China*

[3]*Department of Physics, University of Science & Technology of China, Hefei 230026, China*

[4]*Electron Microscopy Laboratory, School of Physics, Peking University, Beijing 100871, China*

[†]These authors contributed equally to this work.

**Corresponding author: leifu@whu.edu.cn*

Contents

Supplementary Information 1
1. Materials 3
2. Interlayer spacing expansion of Ce–MoS_2 4
3. Interlayer spacing compression of Pt, Ce–MoS_2 with Pt intercalating 5
4. Interlayer spacing expansion of Pt, Ce–MoS_2 with the decrease of Pt concentration via an eluting process.. 6
5. CV curves during the electrochemical intercalation and elution process. 7
6. TEM characterization of the Ce–MoS_2 crystal 8
7. HAADF–STEM image of Pt, Ce–MoS_2 at a larger scale 9
8. The identification of Ce atoms in MoS_2 10
9. HAADF–STEM image of Pt, Ce–MoS_2 after Pt elution 11
10. Endurance of the 2D MoS_2 12
11. HAADF–STEM image of eluted Pt, Ce–MoS_2 after 5 cycles. 13
12. SEM image of eluted Pt, Ce–MoS_2 after 5 cycles 14
13. XPS analysis for identifying the valance state of Ce and Pt in Pt, Ce–MoS_2 15
14. TEM characterizations and EDS mapping of Pt, Ce–MoS_2 16
15. The variation of the XPS spectra of Mo 3d core level derived from the Ce–MoS_2 samples with the increase of Ce doping concentration 17
16. Stacking model and HAADF−STEM image of intrinsic MoS_2 18
17. Stacking model and HAADF−STEM image of Ce−MoS_2. 19
18. Interlayer spacing expansion of MoS_2 with direct intercalation of Pt 20
19. XPS analysis for identifying the valance state of Pt in the eluting Pt, Ce–MoS_2 intermediate 21
20. Interlayer spacing change of WS_2 with the introduction of Ce and Pt via electrochemical process 22
21. HAADF–STEM image of Pt, Ce–WS_2 at a large range. 23
22. HAADF–STEM image of eluted Pt, Ce–WS_2 at a large range 24
23. Interlayer spacing change of MoS_2 with the introduction of Pd and Pt via electrochemical process 25
24. HAADF–STEM image of Pt, Pd–MoS_2 at a large range. 26
25. HAADF–STEM image of eluted Pt, Pd–MoS_2 at a large range 27
26. PL spectra of MoS_2 with different Ce concentration 28
27. Calculated band gap evolution with interlayer spacing of AA stacking MoS_2. 29
28. Calculated band structure of Ce−MoS_2 with same interlayer spacing with Pt, Ce−MoS_2 30
29. The fitting results of the EXAFS spectra of Pt, Ce–MoS_2 31
30. Interlayer spacing evolution of MoS_2 after introducing Ce and Pt by DFT calculation 32
31. Pt atom absorption energy by DFT calculation 33
32. Calculated interlayer spacing and band gap 34
Supplementary References 35

1. Materials

Ammonium molybdate tetrahydrate ($(NH_4)_6Mo_7O_{24}\cdot 4H_2O$, 99.0%), thiourea ($CH_4N_2S$, 99.0%) and sulfuric acid (H_2SO_4, 98.08%) were purchased from Sinopharm Chemical Reagent Co., Ltd. Anhydrous cerium chloride ($CeCl_3$, 99.99%) and titanium (Ti) foil (99.6%) were purchased from Alfa Aesar. Ammonium Metatungstate ($(NH_4)_6H_2W_{12}O_{40}\cdot xH_2O$, 99.5%), Thioacetamide (CH_3CSNH_2, 98.0%) are purchased from Aladdin. The deionized water was purified by using Arium bagtank 50 (Sartorius).

2. Interlayer spacing expansion of Ce–MoS_2

The interlayer spacing of MoS_2 exhibits a linear increase with the Ce concentration due to the gradual weakening of interlayer coupling caused by the electron transferring to the S–Mo anti-bonding orbitals, as shown in Supplementary Figure 1. When the concentration of Ce increases from 0% to 0.995%, the interlayer spacing of MoS_2 changes from 6.232 ± 0.024 Å to 6.546 ± 0.039 Å.

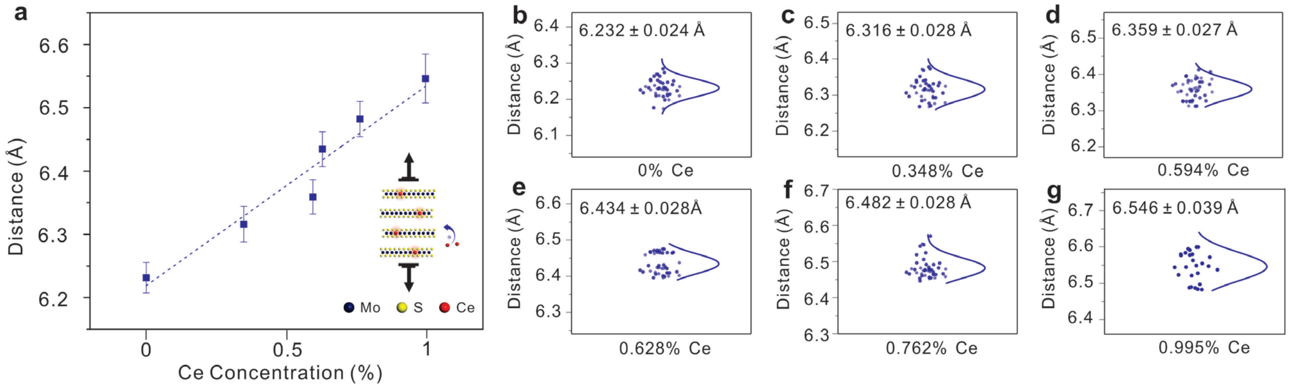

Supplementary Figure 1 | Interlayer spacing expansion of Ce–MoS_2. a, Interlayer spacing regulation of MoS_2 with the increase of the Ce doping concentration. A good linear relationship between the interlayer spacing of Ce–MoS_2 and the Ce doping concentration can be observed. Error bars represent standard deviation over 26 independent replicates at least. **b–g**, The statistical distribution of interlayer spacing of Ce–MoS_2 with different Ce doping concentrations corresponding to those in **a**.

3. Interlayer spacing compression of Pt, Ce–MoS_2 with Pt intercalating

The monodispersed Pt atoms were intercalated into the interlamination of Ce−MoS_2 via an electrochemical reaction to reduce the interlayer spacing. When the concentration of Pt increases from 0% to 2.31%, the interlayer spacing of MoS_2 changes from 6.546 ± 0.039 Å to 5.792 ± 0.038 Å (Supplementary Figure 2).

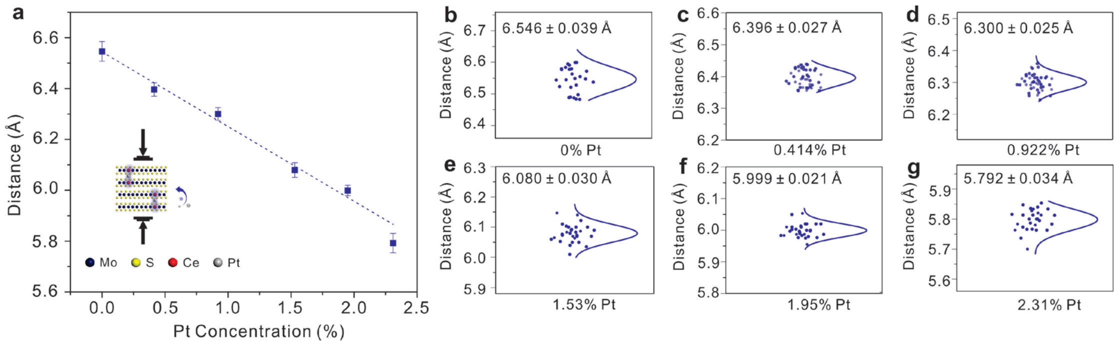

Supplementary Figure 2 | Interlayer spacing compression of Pt, Ce–MoS_2 with Pt intercalating. a, Interlayer spacing regulation of Pt, Ce–MoS_2 with the increase of the Pt intercalating concentration. A good linear relationship between the interlayer spacing of Pt, Ce–MoS_2 and the Pt intercalating concentration can be observed. Error bars represent standard deviation over 26 independent replicates at least. **b–g**, The statistical distribution of interlayer spacing of Pt, Ce–MoS_2 with different Pt intercalating concentrations corresponding to those in **a**.

4. Interlayer spacing expansion of Pt, Ce–MoS_2 with the decrease of Pt concentration via an eluting process

The single Pt atoms that anchored on Ce–MoS_2 can be electrochemically eluted and the interlayer spacing will get expanded. When the concentration of Pt decreases from 2.31% to 0.14%, the interlayer spacing of layered Ce–MoS_2 gets recovered to 6.494 ± 0.032 Å (Supplementary Figure 3).

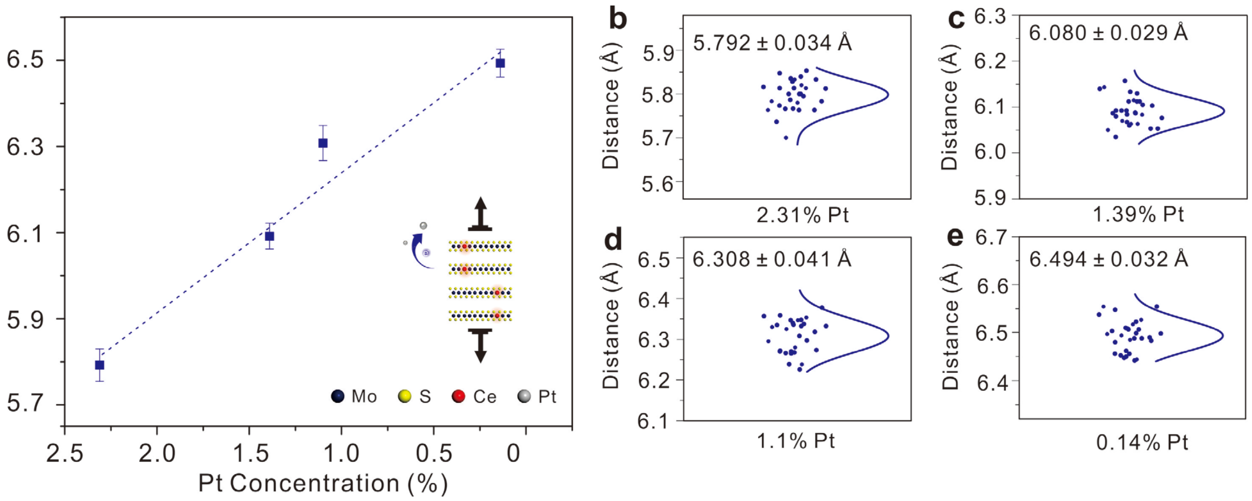

Supplementary Figure 3 | Interlayer spacing expanding of Pt, Ce–MoS_2 with the decrease of Pt concentration via an eluting process. a, Interlayer spacing regulation of Pt, Ce–MoS_2 with decreasing Pt intercalation concentration. A good linear relationship between the interlayer spacing of Pt, Ce–MoS_2 and the Pt concentration can be observed, delivering the slope with the absolute value similar to that in the Pt intercalating situation. Error bars represent standard deviation over 30 independent replicates. **b–e**, The statistical distribution of interlayer spacing of Pt, Ce–MoS_2 with different Pt concentrations corresponding to those in **a**.

5. The CV curves during the electrochemical intercalation and elution process

The cyclic voltammetry (CV) curves accompanying the intercalating and eluting processes of Pt are presented in Supplementary Figure 4. Supplementary Figure 4a shows the polarization curves of the Pt intercalating process. The increasing current within the voltage range means that the catalytic activity of the hydrogen evolution reaction (HER) is increasing, which corresponds to the Pt intercalation, considering that Pt can serve as the active site of HER reactivity[1]. For elution process, the CV curve is shown in Supplementary Figure 4b. The current is decreasing, which means the elution of Pt atoms from host materials.

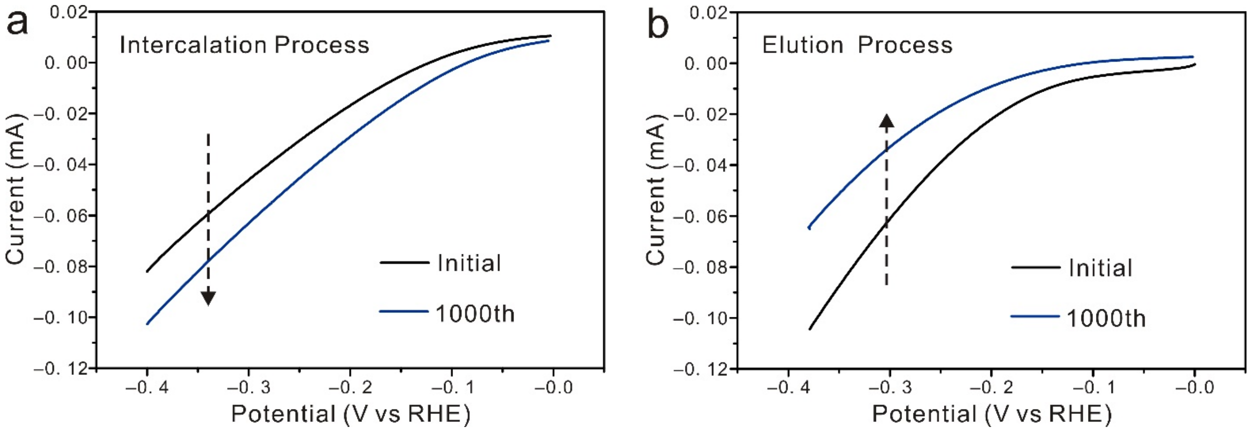

Supplementary Figure 4 | The intercalation and elution of Pt atoms during the electrochemical process. **a**, The CV curve of electrochemical intercalation process **b,** The CV curve of electrochemical elution process.

6. TEM characterization of the Ce–MoS_2 crystal

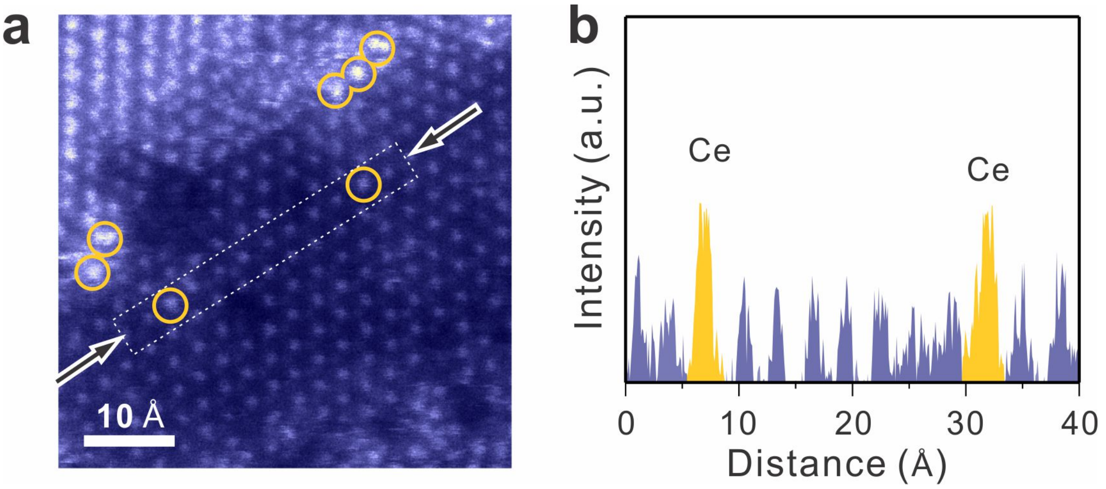

Supplementary Figure 5 | High angle annular dark field scanning transmission electron microscopy (HAADF–STEM) image of Ce–MoS_2 to show the monoatomic dispersion of Ce in the MoS_2. a–b, The HAADF–STEM images of Ce–MoS_2. Ce atoms are marked by yellow circles. Those Ce atoms either locate at the edge of each layer MoS_2 or occupy the Mo sites in the MoS_2 plane. **b**, Line intensity profiles in the selected regions in **a**.

7. HAADF–STEM image of Pt, Ce–MoS_2 at a larger scale

It is found that Pt atoms usually existed in accompany with Ce atoms, implying their diatomic pair relationship.

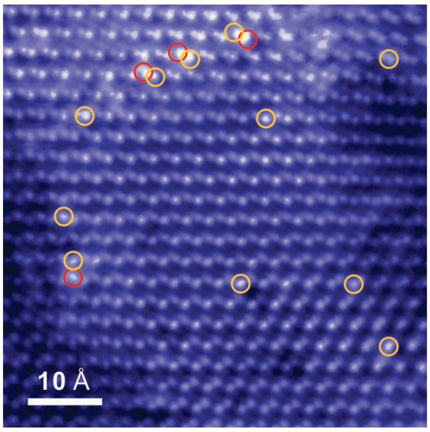

Supplementary Figure 6 | HAADF–STEM image of Pt, Ce–MoS_2. The embedded Ce atoms in the occupation of Mo sites are marked by yellow circles and the intercalated Pt atoms are marked by red circles.

8. The identification of Ce atoms in MoS_2

Supplementary Figure 7a exhibits the energy dispersive X-ray (EDX) spectrum of Pt, Ce–MoS_2, in which the peaks attributed to Pt and Ce can be clearly observed. The statistical distribution of the annular dark-field scanning (ADF) peak intensity corresponding to the Mo atoms and the brighter atoms that are in the occupation of Mo sites and marked with yellow circles (Figure 2a) is shown in Supplementary Figure 7. Here, for better comparison, the ADF peak intensity was normalized. The intensity of Mo atoms was set to be 1. The ADF intensity of the atoms in yellow circles shows a centralized distribution and the central value is about 1.75. The intensity ratio of these two kinds of atoms is in consistence with the value of $Z_{Ce}^{1.7}/Z_{Mo}^{1.7}$ (= 1.73). Therefore, those heteroatoms on Mo sites are thought to be Ce[2].

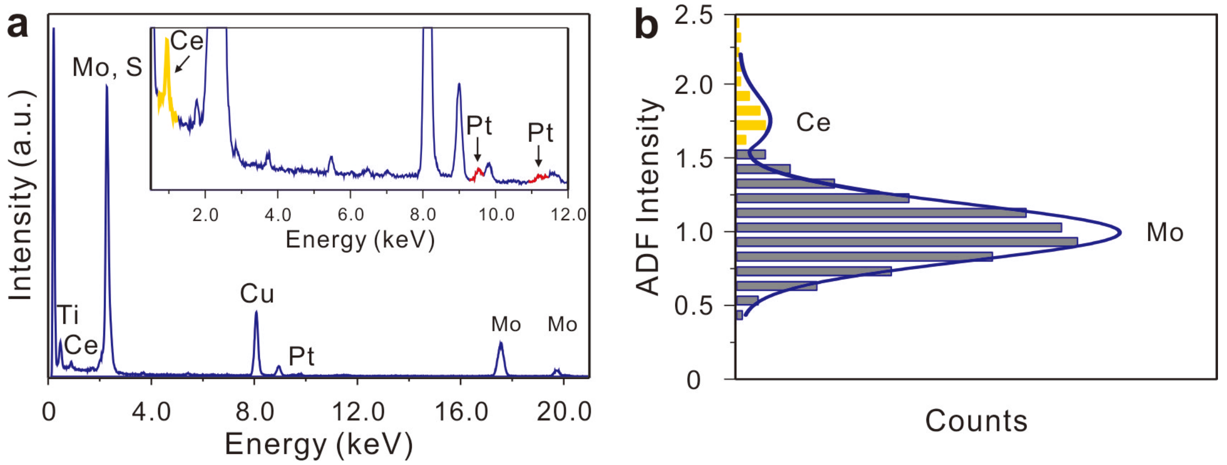

Supplementary Figure 7 | The identification of Ce atoms in MoS_2. a, EDS of Pt, Ce–MoS_2. **b**, Histogram of the ADF intensity distribution of Mo and Ce in selected planar MoS_2 ADF images. Here, for better comparison, the ADF peak intensity was normalized. The intensity of Mo atoms was set to be 1. The statistical ADF intensities of Mo and Ce atoms are 1 and 1.75, respectively.

9. HAADF–STEM image of Pt, Ce–MoS_2 after Pt elution

As seen in Supplementary Figure 8, there are only Ce atoms in MoS_2, which were identified by the intensity ratio. The Pt atoms can be eluted by the electrochemical method, indicating the diatomic pair relationship can be built reversibly.

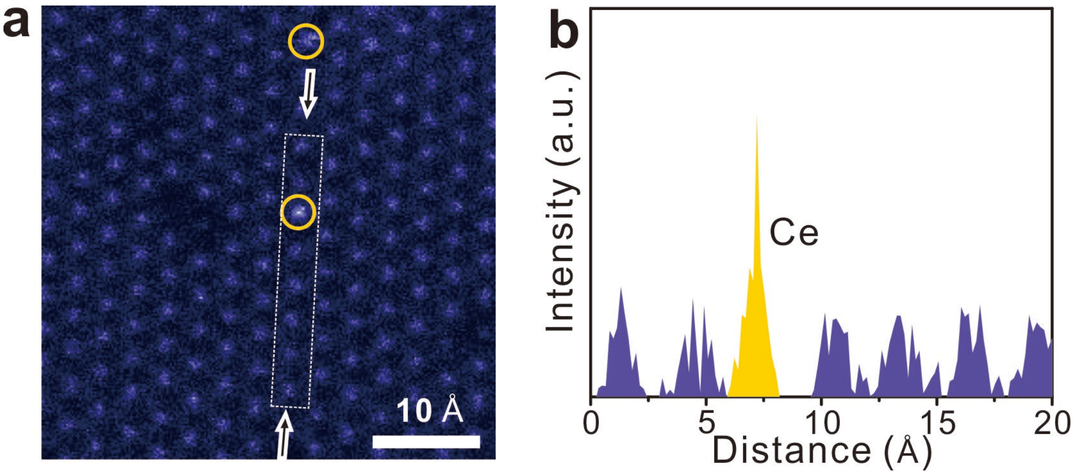

Supplementary Figure 8 | HAADF–STEM image of eluted Pt, Ce–MoS_2. a, The HAADF–STEM images of Pt, Ce–MoS_2 after Pt elution. Ce atoms are marked by yellow circles. The Ce atom occupies the Mo site in the MoS_2 plane. **b**, Line intensity profile in the selected regions marked by a white dashed box in **a**.

10. Endurance of the 2D Pt, Ce–MoS_2 with reversible interlayer spacing

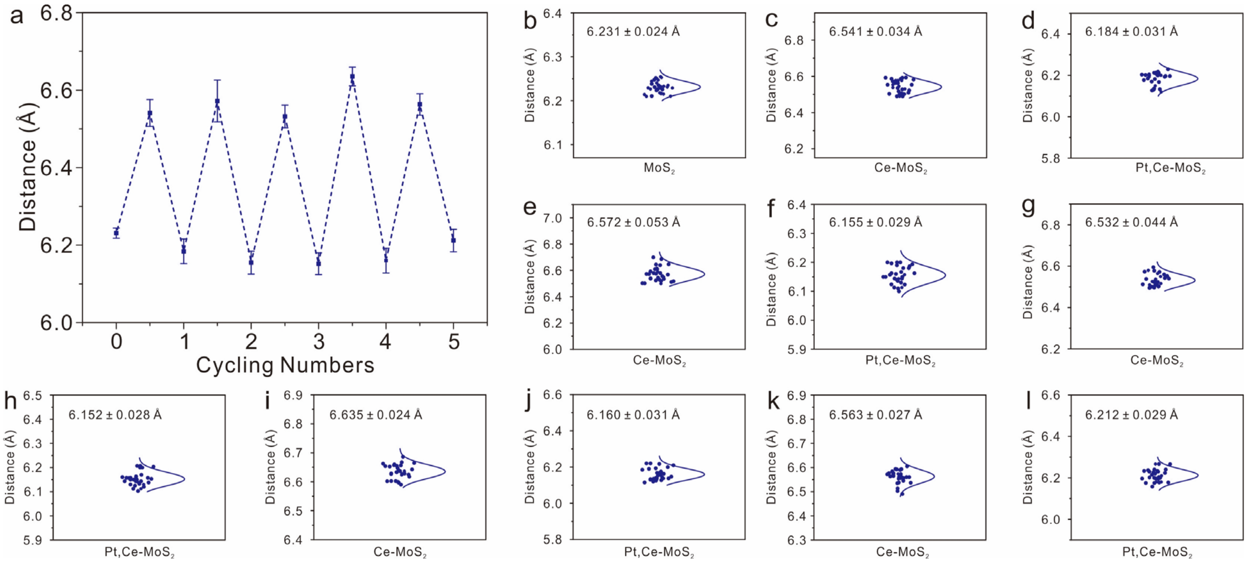

Supplementary Figure 9 | Interlayer spacing change with the intercalation and elution of Pt. a, Interlayer spacing regulation of Pt, Ce–MoS_2 with the intercalation and elution of Pt. Error bars represent standard deviation over 30 independent replicates at least. **b–l**, The statistical distribution of interlayer spacing of Pt, Ce–MoS_2 with the intercalation and elution of Pt corresponding to those in **a**.

11. HAADF–STEM image of eluted Pt, Ce–MoS_2 after 5 cycles

As seen in Supplementary Figure 10, there are no defects in MoS_2 after Pt elution, indicating that our diatomic pair strategy is bidirectional and reversible.

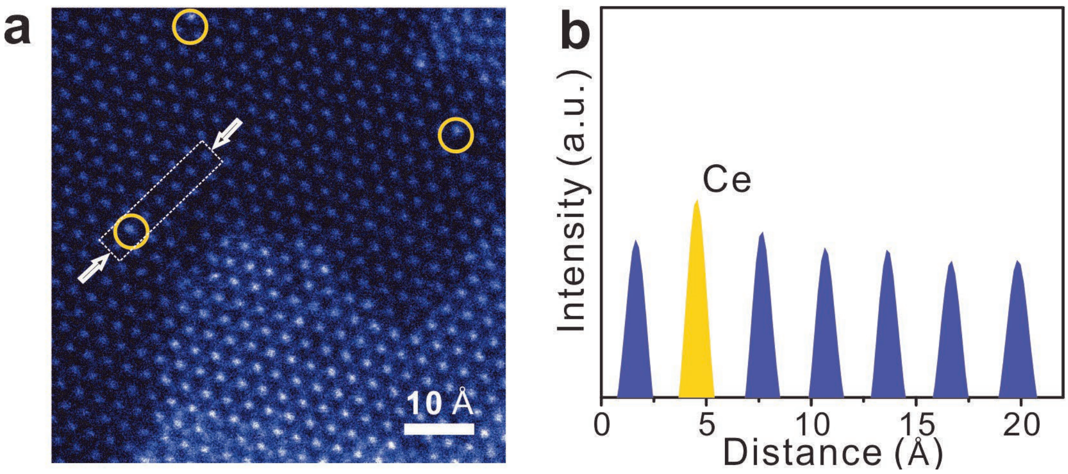

Supplementary Figure 10 | HAADF–STEM image of eluted Pt, Ce–MoS_2 after 5 cycles. a, The HAADF–STEM images of eluted Pt, Ce–MoS_2 after 5 cycles. Ce atoms are marked by yellow circles. Those Ce atoms occupy the Mo sites in the MoS_2 plane. **b**, Line intensity profiles in the selected regions marked by a white dashed box in **a**.

12. SEM image of eluted Pt, Ce–MoS_2 after 5 cycles

In addition, Ce−MoS_2 undergoing five intercalation and elution process maintains a nanoflower morphology, which shows no obvious changes compared with the initial state.

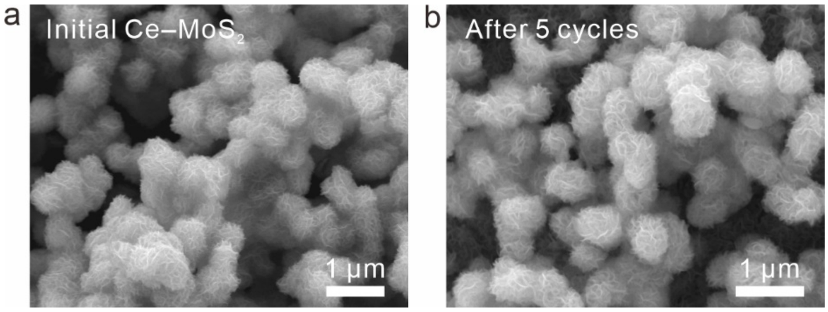

Supplementary Figure 11 | The morphology of Ce−MoS_2. a, intrinsic Ce−MoS_2. **b**, Ce−MoS_2 after 5 cycles.

13. XPS analysis for identifying the valance state of Ce and Pt in Pt, Ce–MoS_2

As seen in Supplementary Figure 12, the X-ray photoelectron spectroscopy (XPS) spectrum of Ce 3d showed six main peaks labeled as v, v'', v''' u, u'' and u'''. Those are the characteristic peaks of Ce^{4+}(Reference 3). However, the existence of v' means there is a small amount of Ce^{3+}. Meanwhile, the XPS spectrum of Pt 4f showed two peaks at 72.9 eV and 76.2 eV, which are in consistence with the characteristic peak positions of Pt–S bonds[4].

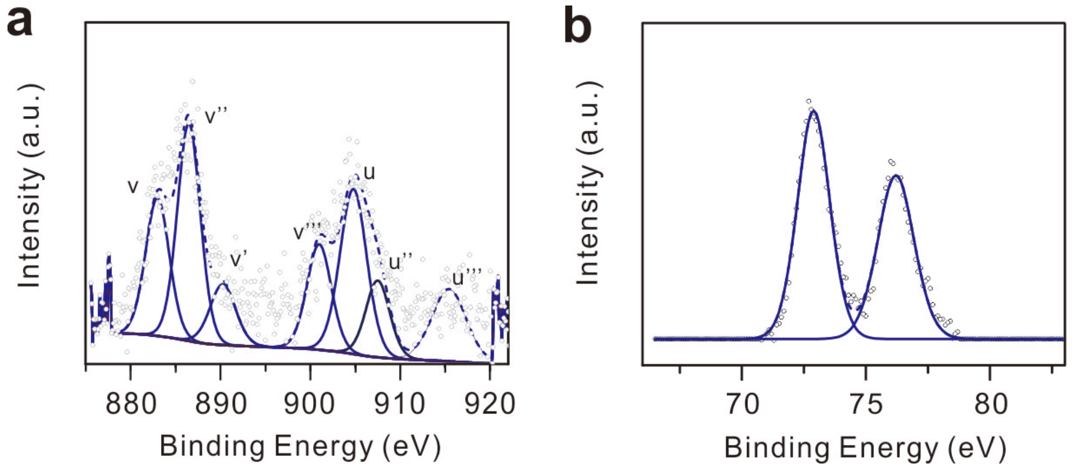

Supplementary Figure 12 | XPS analysis for identifying the valance state of Ce and Pt in Pt, Ce–MoS_2. a, XPS spectrum of Ce. **b**, XPS spectrum of Pt.

14. TEM characterizations and EDS mapping of Pt, Ce–MoS_2

TEM image and the related EDX spectroscopy mapping analysis also showed that no aggregated Pt clusters formed on MoS_2 no matter for in-plane or out-of-plane regions (Supplementary Figure 13).

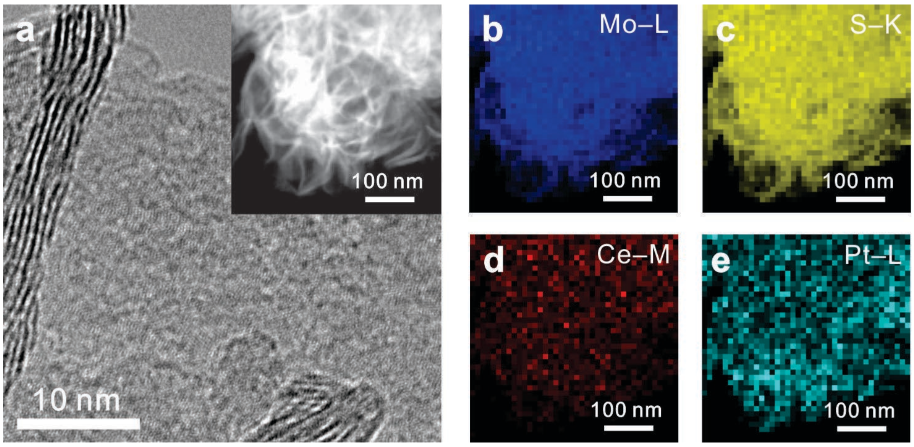

Supplementary Figure 13 | TEM characterizations and EDX mapping of Pt, Ce–MoS_2. a, TEM image of Pt, Ce–MoS_2. No aggregated atom clusters can be observed on the plane or in the interlamination of MoS_2. The inset is the low-magnification STEM image of Pt, Ce–MoS_2. **b–e**, EDS mappings of Mo–L, S–K, Ce–M and Pt–L corresponding to the region in **a**. All the elements are uniformly distributed across the Pt, Ce–MoS_2.

15. The variation of the XPS spectra of Mo 3d core level derived from the Ce–MoS_2 samples with the increase of Ce doping concentration

XPS spectrum of core Mo 3d exhibits two different valence states due to the intrinsic defects and impurities, which are high-state Mo and low-state Mo. With the increase of Ce concentration, the binding energy of Mo 3d exhibits a downshift and the ratio of the high-state Mo to the low-state Mo gradually increases, suggesting the significant electron transferring to the host MoS_2 and the in-plane doping of Ce ions to the host MoS_2. Thus, the anti-orbitals of S–Mo in adjacent MoS_2 layers would repulse each other, leading to a larger equilibrium distance due to the introducing of extra negative charge.

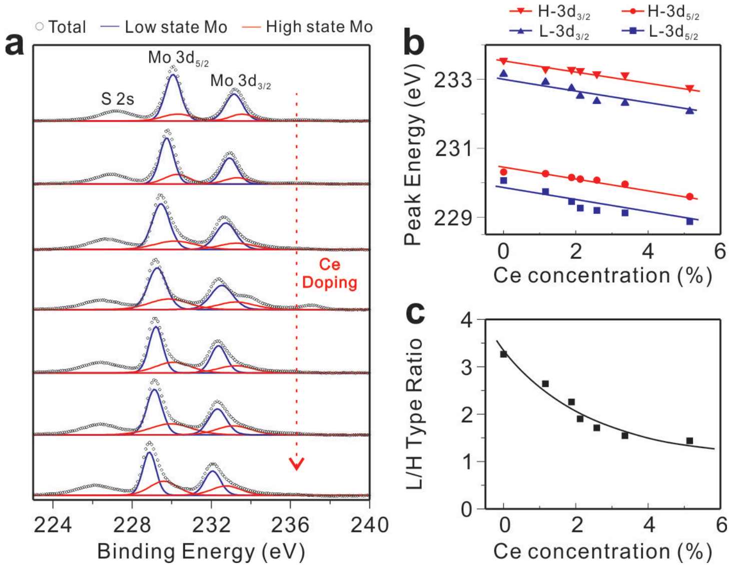

Supplementary Figure 14 | The variation of the XPS spectra of Mo 3d core level derived from the Ce–MoS_2 samples with the increase of Ce doping concentration. a, XPS spectra of Mo 3d core level derived from the Ce–MoS_2 samples with the increase of Ce doping concentration. **b**, The peak position variation of high-state Mo $3d_{3/2}$, low-state Mo $3d_{3/2}$, high-state Mo $3d_{5/2}$ and low-state Mo $3d_{5/2}$ with the increase of Ce concentration. **c**, The ratio evolution of high-state Mo to low-state Mo with the increase of Ce concentration.

16. Stacking model and HAADF−STEM image of intrinsic MoS_2

According to the HAADF−STEM image, we constituted an atom stacking model of intrinsic MoS_2. The calculated interlayer spacing of this stacking MoS_2 is 6.22 Å, which is close to that of experiment (6.232 ± 0.024 Å).

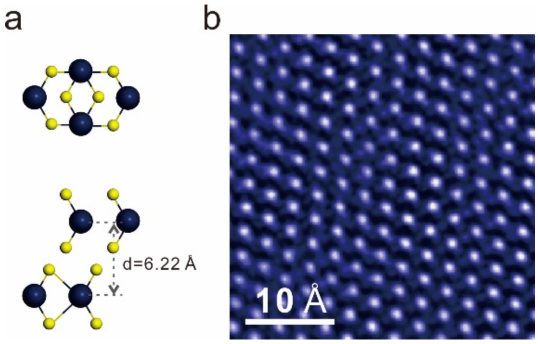

Supplementary Figure 15 | Stacking model and HAADF−STEM image of intrinsic MoS_2 a, stacking model of intrinsic MoS_2. **b**, HAADF−STEM image of intrinsic MoS_2.

17. Stacking model and HAADF−STEM image of Ce-MoS_2

The introduction of Ce ions will lead to the change of MoS_2 stacking structure, which is manifested in Supplementary Figure 16. Ce−MoS_2 exhibits AA stacking model, whose calculated interlayer spacing is 6.78 Å, larger than the value of intrinsic MoS_2 (6.22 Å).

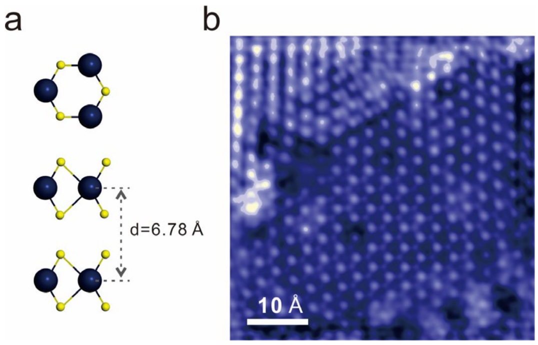

Supplementary Figure 16 | Stacking model and HAADF−STEM image of Ce-MoS_2. a, stacking model of Ce−MoS_2. **b**, HAADF−STEM image of Ce−MoS_2.

18. Interlayer spacing expansion of MoS_2 with direct intercalation of Pt

It can be found that the interlayer spacing of MoS_2 will get expanded with the direct intercalation of Pt without Ce ions (Supplementary Figure 17), consisting with the literature[5]. For one thing, Pt atoms tend to aggregate into nanoparticles without the stabilization of Ce ions, in which the steric effect will result in the interlayer spacing expansion. For another thing, metal atom intercalation will also lead to the expansion of the interlayer spacing inevitably due to the introduction of free electrons that will increase Fermi energy levels and expand the band gaps.

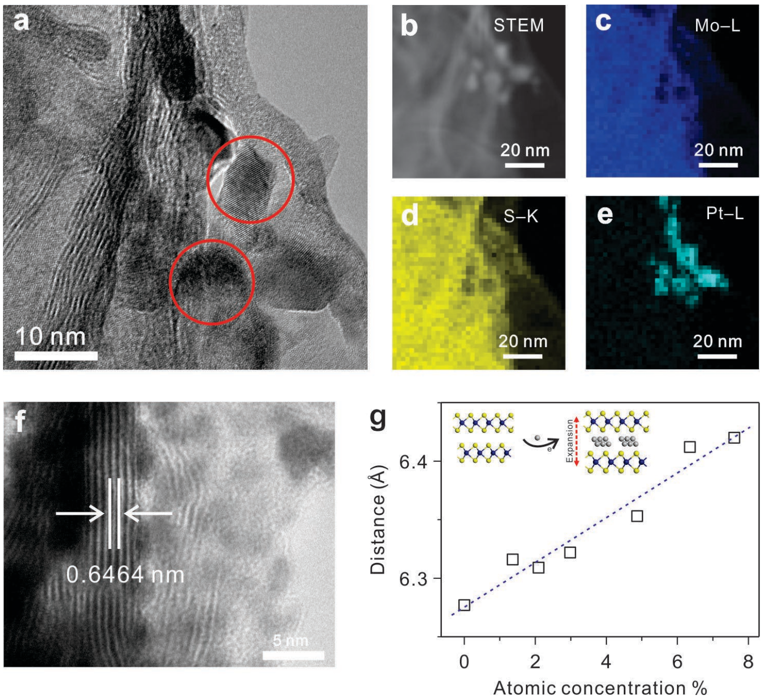

Supplementary Figure 17 | Interlayer spacing expansion of MoS_2 with direct intercalation of Pt. a, TEM image of Pt–MoS_2. Aggregated Pt atom clusters can be clearly observed on the plane or in the interlayer of MoS_2. **b**, Low-magnification STEM image of Pt–MoS_2. **c–e**, EDS mappings of Mo–L, S–K, and Pt–L corresponding to the region in **b**. Mo and S are uniformly distributed across the Ce–MoS_2 while significant aggregation of Pt atom clusters was formed in a local region. **f**, TEM image of layered edges of intrinsic MoS_2 with direct Pt intercalation. The Pt atom clusters between MoS_2 layers will enlarge the interlayer spacing of MoS_2. **g**, Interlayer spacing regulation of MoS_2 via the direct intercalation of Pt.

19. XPS analysis for identifying the valance state of Pt in the eluting Pt, Ce–MoS_2 intermediate

As seen in Supplementary Figure 18, the XPS spectrum of Pt 4f showed four peaks at 72.9 eV, 76.3 eV, 73.6 eV and 77.3 eV, which are in consistence with the characteristic peak positions of Pt–S bonds and Pt−O bonds[4,6]. The XAFS results indicate that there is only Pt−S coordination in Pt, Ce−MoS_2 (Figure 2 and Supplementary Table 1). Such an intermediate may result in the elution of Pt due to that the stronger Pt−O bonds will lead to the breaking of the Pt−S bonds during the HER process[7,8].

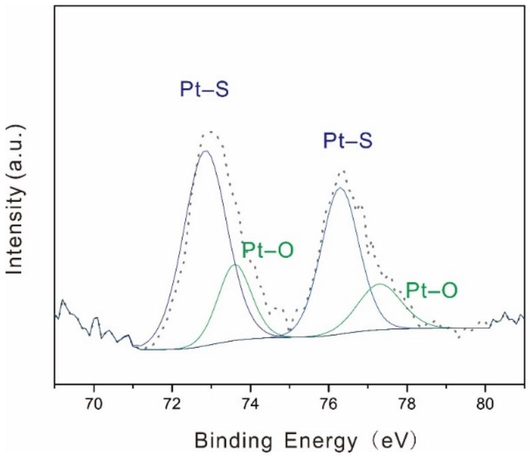

Supplementary Figure 18 | XPS analysis for identifying the valance state of Pt in the eluting Pt, Ce–MoS_2 intermediate.

20. Interlayer spacing change of WS_2 with the introduction of Ce and Pt via electrochemical process

The change of interlayer spacing of WS_2 with the introduction of Ce and Pt is exhibited (Supplementary Figure 19a). The intrinsic interlayer spacing of WS_2 is 6.218 ± 0.021 Å (Supplementary Figure 19b). And it expanded to 6.562 ± 0.035 Å when WS_2 was doped with Ce (Supplementary Figure 19c) and shrank to 6.142 ± 0.025Å after Pt intercalation (Supplementary Figure 19d). The interlayer spacing recovered to 6.520 ± 0.026 Å when the Pt atoms were eluted during the electrochemical process (Supplementary Figure 19e).

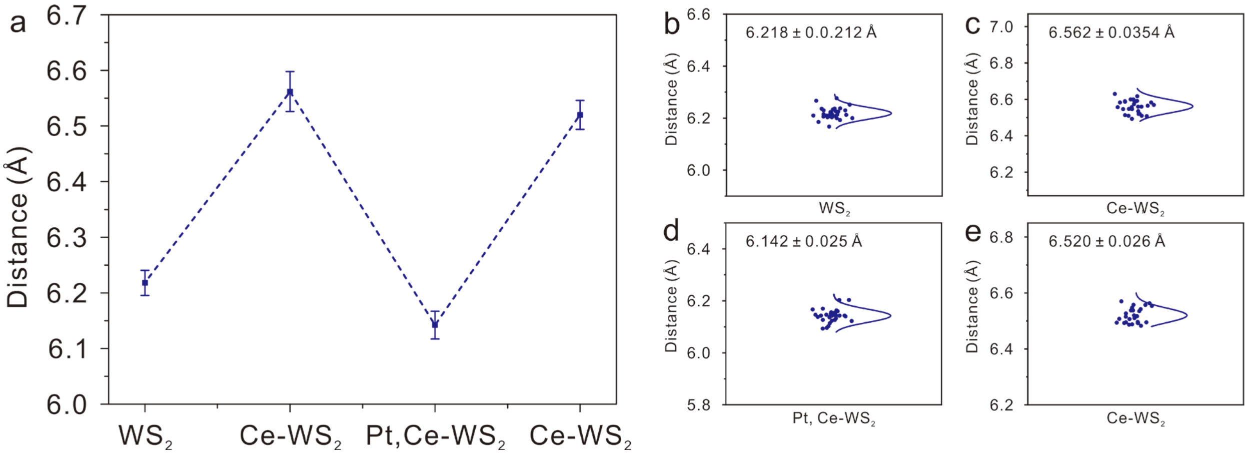

Supplementary Figure 19 | Interlayer spacing change of WS_2 with the introduction of Ce and Pt via electrochemical process. a, Interlayer spacing regulation of WS_2 via Ce doping, Pt intercalating and Pt eluting. Error bars represent standard deviation over 30 independent replicates. **b−e**, The statistical distribution of interlayer spacing of Pt, Ce–WS_2 corresponding to those in **a.**

21. HAADF–STEM image of Pt, Ce–WS_2

It is found that Pt atoms usually existed in accompany with Ce atoms in the WS_2, implying their diatomic pair relationship within WS_2.

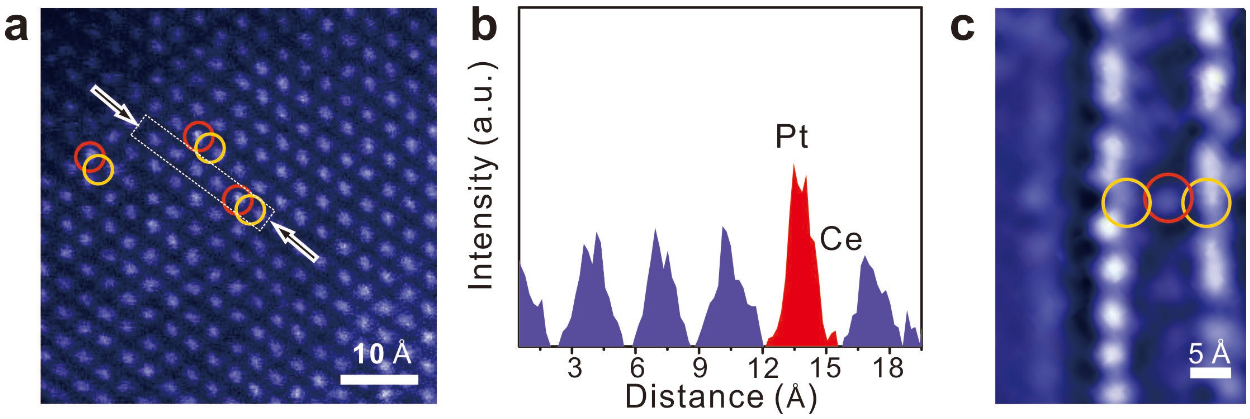

Supplementary Figure 20 | HAADF–STEM image of Pt, Ce–WS_2. a, The HAADF–STEM image of Pt, Ce–WS_2. Ce atoms are marked by yellow circles and Pt atoms are marked by red circles. Those Ce atoms occupy the W sites in the WS_2 plane. **b**, Line intensity profiles in the selected regions marked by a white dashed box in **a. c**, The cross-sectional HAADF–STEM images of Pt, Ce–WS_2 at the edge site.

22. HAADF–STEM image of eluted Pt, Ce–WS_2

As seen in Supplementary Figure 21, there are only Ce atoms in WS_2, which was identified by the intensity ratio. The Pt atoms can be eluted by the electrochemical method, indicating that the diatomic pair relationship in the WS_2 can be built reversibly.

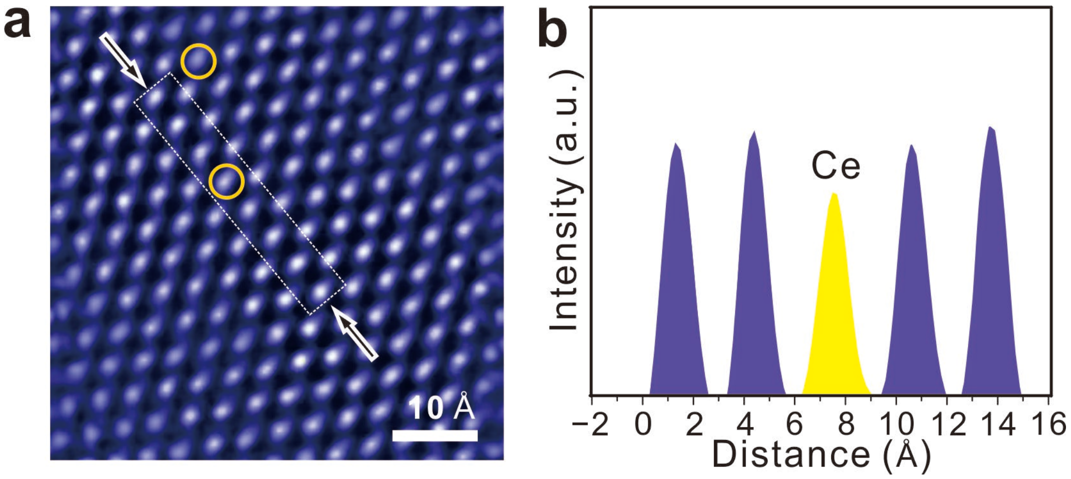

Supplementary Figure 21 | HAADF–STEM image of eluted Pt, Ce–WS_2. a, The HAADF–STEM images of Pt, Ce–WS_2 after Pt elution. Ce atoms are marked by yellow circles. Those Ce atoms occupy the Mo sites in the MoS_2 plane. **b**, Line intensity profiles in the selected regions marked by a white dashed box in **a**.

23. Interlayer spacing change of MoS_2 with the introduction of Pd and Pt via electrochemical process

The change of interlayer spacing of MoS_2 with the introduction of Pd and Pt is exhibited (Supplementary Figure 22a). The intrinsic interlayer spacing of MoS_2 is 6.232 ± 0.024 Å (Supplementary Figure 22b). And it expanded to 6.598 ± 0.031 Å when MoS_2 was doped with Pd (Supplementary Figure 22c) and shrank to 6.199 ± 0.038 Å after Pt intercalation (Supplementary Figure 22d). The interlayer spacing recovered to 6.533 ± 0.032 Å when the Pt atoms were eluted during the electrochemical process (Supplementary Figure 22e).

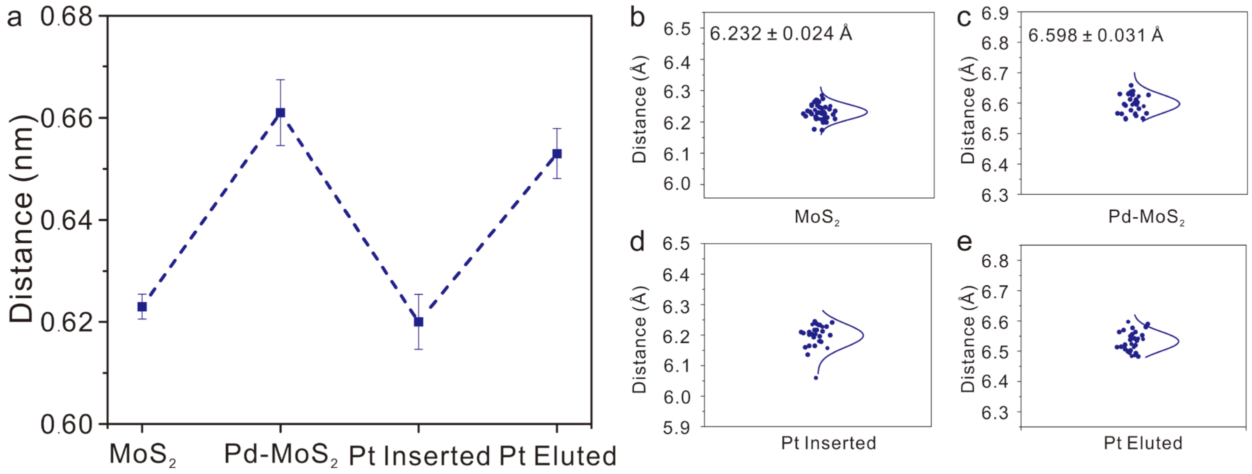

Supplementary Figure 22 | Interlayer spacing change of MoS_2 with the introduction of Pd and Pt via electrochemical process. a, Interlayer spacing regulation of MoS_2 via Pd doping, Pt intercalating and Pt eluting. Error bars represent standard deviation over 40 independent replicates in **b** and 30 independent replicates in **c**–**e**. **b**–**e**, The statistical distribution of interlayer spacing of Pt, Pd–MoS_2 corresponding to those in **a**.

24. HAADF–STEM image of Pt, Pd–MoS_2

It is found that Pt atoms usually existed in accompany with Pd atoms in the MoS_2, implying their diatomic pair relationship.

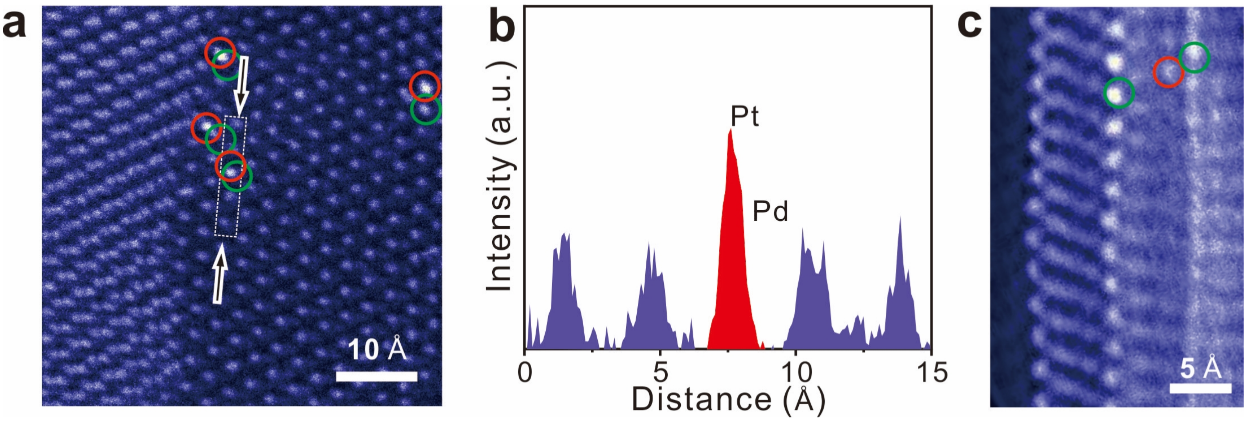

Supplementary Figure 23 | HAADF–STEM image of Pt, Pd–MoS_2. a, The HAADF–STEM image of Pt, Pd–MoS_2. Pd atoms are marked by green circles and Pt atoms are marked by red circles. Those Pd atoms occupy the Mo sites in the MoS_2 plane. **b**, Line intensity profiles in the selected regions marked by a white dashed box in **a. c**, The cross-sectional HAADF–STEM images of Pt, Pd–MoS_2 at the edge site.

25. HAADF–STEM image of eluted Pt, Pd–MoS_2

As seen in Supplementary Figure 24, there are no obviously brighter atoms in MoS_2, indicating that the Pt atoms can be eluted by the electrochemical method. Therefore, the diatomic pair relationship in the MoS_2 can be built reversibly.

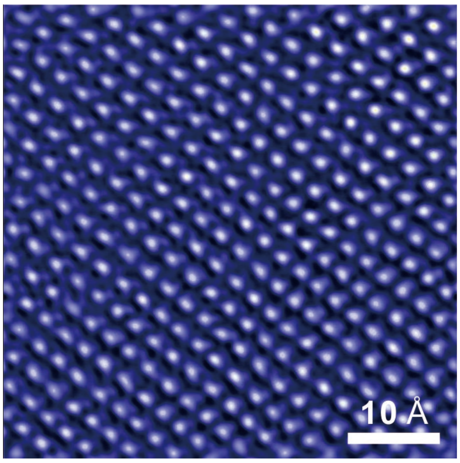

Supplementary Figure 24 | HAADF–STEM image of eluted Pt, Pd–MoS_2.

26. PL spectra of MoS_2 with different Ce concentration

The interlayer spacing of MoS_2 increases with the Ce concentration increasing, resulting that the PL A peak position of the MoS_2 shifts from 668 nm to 658 nm (Supplementary Figure 25). The change of peak position means that the interlayer-spacing-enlarged multi-layer MoS_2 behaved as monolayer-like MoS_2, for which its optical band gap value is 1.899 eV that is close to the value for the band gap of monolayer MoS_2 (1.90 eV) in the reported work[9]. In addition, the intensity of the PL A peak is also greatly enhanced. This interlayer decoupling effect is derived from the weakening of *van der Waals* interaction with the expanding of the interlayer spacing.

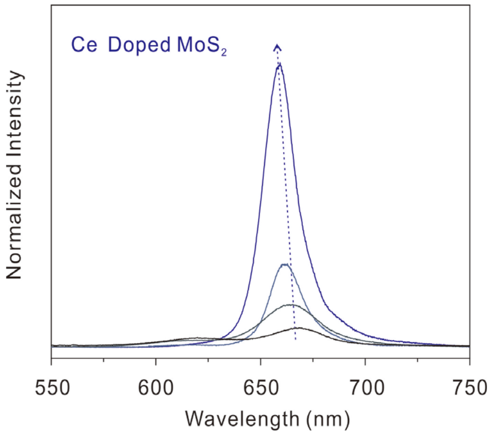

Supplementary Figure 25 | Photoluminescence (PL) spectra of MoS_2 with different Ce concentrations. With the increase of Ce concentration, the interlayer spacing of MoS_2 gets enlarged and the main PL A peak position of the MoS_2 shifts from 668 nm to 658 nm with the gradually enhanced peak intensity.

27. Calculated band gap of AA stacking MoS_2 with the interlayer spacing variation

The band gap evolution with interlayer spacing of AA stacking MoS_2 is calculated, which is shown in Supplementary Figure 26. From Figure 4b, we can extract the band gap of Ce−MoS_2 (1.648 eV), whose interlayer spacing is 6.78 Å. This interlayer spacing related band gap in intrinsic AA stacking MoS_2 is 1.626 eV. Therefore, both the Ce doping and interlayer spacing enlargement will lead to the increasing of the band gap. The calculated results are summarized in Supplementary Table 4.

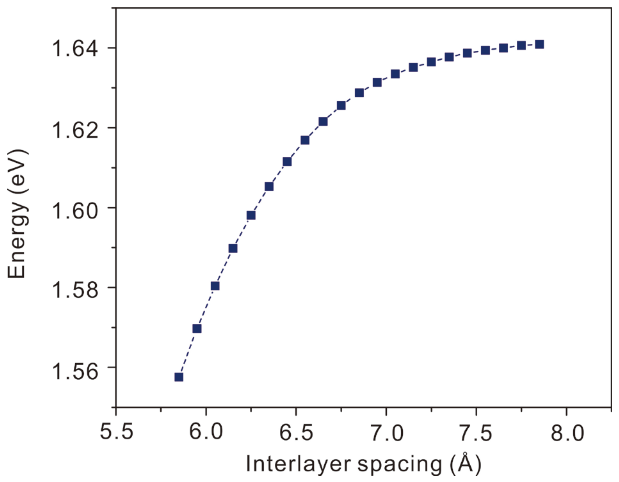

Supplementary Figure 26 | Calculated band gap (E_{CBM}−E_{VBM}) at the K point of intrinsic AA stacking MoS_2 with the interlayer spacing variation.

28. Calculated band structure of Ce−MoS_2 with the same interlayer spacing with that of Pt, Ce−MoS_2

We fixed the interlayer spacing of AA stacking Ce−MoS_2 and AA stacking Pt, Ce−MoS_2 and found that the Pt insertion would result in the decreasing of the band gap. When the interlayer spacing of AA stacking Ce−MoS_2 is set to be 6.61 Å, which is equal to that of AA stacking Pt, Ce−MoS_2. The band gap of Ce−MoS_2 and Pt, Ce−MoS_2 is 1.592 eV and 1.577 eV (Figure 4c), respectively. As a comparison, the band gap of Ce−MoS_2 without fixed interlayer spacing is 1.648 eV (Figure 4b). Therefore, the intercalation of Pt will decrease the band gap of MoS_2 intrinsically, when the effect of layer spacing is not considered. However, the Pt intercalation is not the main contributor of the band gap decreasing of Pt, Ce−MoS_2. All calculated results are listed at Supplementary Table 4.

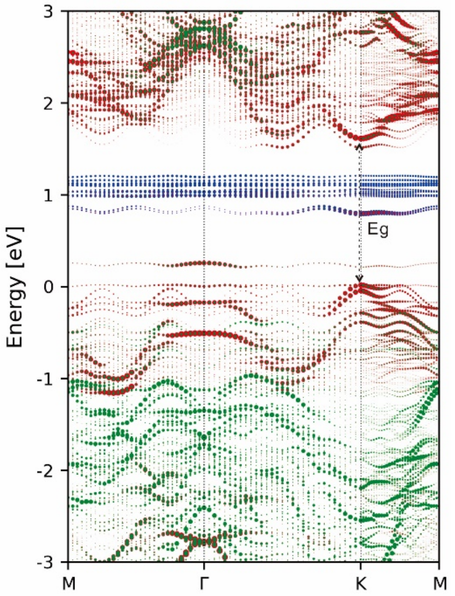

Supplementary Figure 27 | Calculated unfolded band structure of Ce–MoS_2 with the interlayer spacing fixed by that of Pt, Ce−MoS_2.

29. The fitting results of the EXAFS spectra of Pt, Ce–MoS_2

In order to investigate the structural information of the Pt, Ce−MoS_2, extended X-ray absorption fine structure (EXAFS) measurements at Pt L_3-edge were carried out. The data ranges used for data fitting in k space (Δk) and R space are 3.45–9.9 and 1.25–2.3 Å, respectively.

Supplementary Table 1. The fitting results of the EXAFS spectra of Pt, Ce–MoS_2.

Shell	Bond length (Å)	Coordination number	E_0 shift (eV)	Mean squared displacement (Å^2)
Pt–S	2.32	4.95 ± 0.57	9.07	0.00523

30. Interlayer spacing evolution of MoS_2 after introducing Ce and Pt by DFT calculation

In order to further investigate the role played by Ce and Pt in the regulation of MoS_2 interlayer spacing, the DFT calculations are implemented.

Supplementary Table 2. Calculated interlayer spacing of intrinsic MoS_2, AA stacking Ce−MoS_2 and AA stacking Pt, Ce−MoS_2.

	Intrinsic MoS_2	AA stacking Ce−MoS_2	AA stacking Pt, Ce−MoS_2
Interlayer spacing (Å)	6.22	6.78	6.61

31. Absorption energy of Pt atom on Ce-MoS_2 by DFT calculation

The strong interaction between Ce ions and Pt atoms provides us an accessible approach to dispersing and stabilizing Pt single atoms. For Ce–MoS_2, we calculated the absorption energy of Pt atoms on Ce–MoS_2 at different sites. The Supplementary Figure 28 show the five situations.

We artificially set the absorption energy corresponding to Supplementary Figure 28a at zero and the other calculated absorption energy values are listed in Supplementary Table 3, from which we can draw the conclusion that the dissolved Pt atom is inclined to intercalate into the interlamination of Ce–MoS_2 instead of absorbing at surface.

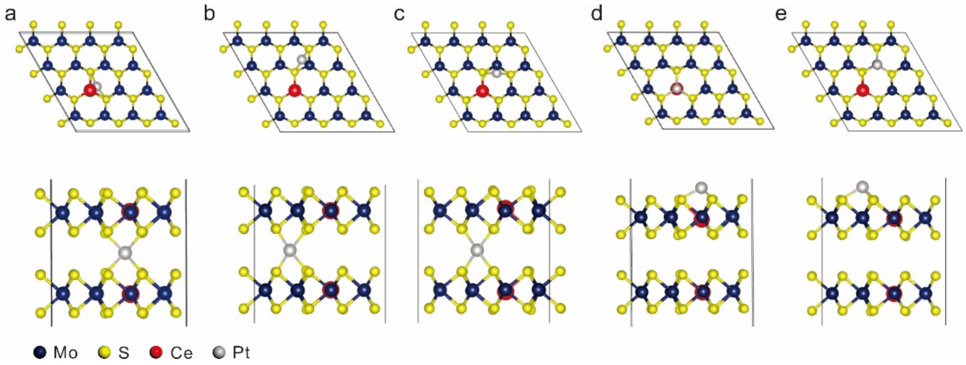

Supplementary Figure 28 | Different situations corresponding to the absorption of Pt single atom on Ce−MoS_2 at different sites. Top, top view; Bottom, side view.

Supplementary Table 3. Relative absorption energy of Pt atom on Ce−MoS_2 at different sites, where the absorption energy of situation **a** is set as energy reference.

	Situation a	Situation b	Situation c	Situation d	Situation e
ΔE (ads, eV)	0	−0.170587	0.538584	2.193983	2.728722

32. Calculated interlayer spacing and the corresponding band gap

Supplementary Table 4. Calculated interlayer spacing and the corresponding band gap.

	AA stacking MoS_2	AA stacking Ce−MoS_2	AA stacking Ce−MoS_2 with defined interlayer spacing	AA stacking Pt, Ce−MoS_2
Interlayer spacing (Å)	6.78	6.78	6.61	6.61
Band gap (eV)	1.626	1.648	1.592	1.577

Supplementary References

1 Qi, K. *et al.* Single-atom cobalt array bound to distorted 1T MoS_2 with ensemble effect for hydrogen evolution catalysis. *Nat. Commun.* **10**, 5231 (2019).

2 Krivanek, O. L. *et al.* Atom-by-atom structural and chemical analysis by annular dark-field electron microscopy. *Nature* **464**, 571−574 (2010).

3 Lenormand, F. *et al.* Photoemission on 3d-core levels of cerium: an experimental and theoretical investigation of the reduction of cerium dioxide. *Solid State Commun.* **71**, 885−889 (1989).

4 Dembowski, J., Marosi, L. & Essig, M. Platinum sulfide by XPS. *Surf. Sci. Spec.* **2**, 104−108 (1993).

5 Chen, Z. *et al.* Interface confined hydrogen evolution reaction in zero valent metal nanoparticles-intercalated molybdenum disulfide. *Nat. Commun.* **8**, 14548 (2017).

6 Bancroft, G. M. *et al.* ESCA study of sputtered platinum films. *Anal. Chem.* **47**, 586−588 (1975).

7 Liu, Y. *et al.* Low overpotential in vacancy-rich ultrathin $CoSe_2$ nanosheets for water oxidation. *J. Am. Chem. Soc.* **136**, 15670−15675 (2014).

8 Zhang, J. *et al.* Single platinum atoms immobilized on an MXene as an efficient catalyst for the hydrogen evolution reaction. *Nat. Catal.* **1**, 985−992 (2018).

9 Mak, K. F., Lee, C., Hone, J., Shan, J. & Heinz, T. F. Atomically thin MoS_2: A new direct-gap semiconductor. *Phys. Rev. Lett.* **105**, 13680 (2010).
